# Recent Change in Anhedonia, Major Depression and Low-Grade Inflammation: Dangerous Liaisons? A Study Based on a Cohort Referred for Polysomnography

**DOI:** 10.3390/medicina61122125

**Published:** 2025-11-28

**Authors:** Véronique Bernier, Anaïs Mungo, Camille Point, Benjamin Wacquier, Gwenolé Loas, Matthieu Hein

**Affiliations:** 1 Service de Psychiatrie, CHU Brugmann, Université Libre de Bruxelles (ULB), 1020 Brussels, Belgium; veronique.bernier@ulb.be (V.B.); camille.point@chu-brugmann.be (C.P.); 2 Laboratoire de Psychologie Médicale et Addictologie (ULB312), Université Libre de Bruxelles (ULB), 1020 Brussels, Belgium; 3 Faculté de Médecine, Université Libre de Bruxelles (ULB), 1070 Brussels, Belgium; gwenole.loas@ulb.be; 4 Centre Hospitalier Le Domaine-ULB, Service de Psychiatrie, Université Libre de Bruxelles (ULB), 1420 Braine l’Alleud, Belgium; anais.mungo@ulb.be; 5 Service de Psychiatrie, Hôpital Universiatire de Bruxelles, Université Libre de Bruxelles (ULB), 1070 Brussels, Belgium

**Keywords:** major depressive disorder, anhedonia, inflammation

## Abstract

*Background and Objectives:* Anhedonia is a core symptom of major depressive disorder (MDD) and worsens its prognosis. Inflammation has been associated with MDD, contributing to the severity of this pathology despite no clear clinical guidance on whether it should be integrated into the diagnosis or treatment of the MDD symptomatology. Notably, the neural basis of anhedonia is associated with alterations in the reward neural circuit, where inflammation may also interfere. In this study, we investigate whether recent change in anhedonia was associated with low-grade inflammation (defined as C-Reactive Protein levels between 3 and 10 mg/L) in MDD subjects. *Materials and Methods:* A retrospective study was conducted on 496 MDD subjects and drawn from the database of the sleep laboratory. Recent change in anhedonia was assessed via the Anhedonia Subscale of the 21-items Beck Depression Inventory (BDI-II), with scores > 3 indicating its presence. Recent change in anhedonia was defined as the recent onset or worsening of anhedonia complaints within the past 2 weeks. Anxiety and sleep disturbances were also evaluated and inflammatory status was determined based on CRP levels. *Results:* After adjusting for the main confounding factors, the multivariate logistic regression confirms a clear association between recent change in anhedonia and low-grade inflammation, thereby contributing to a detrimental context underlying the symptom. *Conclusions:* A better understanding of anhedonia in the context of inflammation could enable treatment adjustments and improve the poor prognosis of anhedonia-type depression.

## 1. Introduction

Anhedonia is a cardinal symptom of major depressive disorder (MDD) that is characterized by an inability to feel positive emotions during life situations previously considered pleasant [1]. However, in MDD subjects, anhedonia complaints may be associated with the occurrence of multiple negative psychological and somatic consequences. Indeed, compared to subjects without anhedonia, the severity of major depressive episodes is generally higher in subjects with anhedonia [2]. In addition, the clinical course of MDD subjects with anhedonia appears to be less favorable than that of MDD subjects without anhedonia, following the occurrence of increased suicidality and poorer life quality [3,4]. Concerning the impact of anhedonia complaints on the response to antidepressant treatment, it has been demonstrated that the treatment time required to reach remission was longer in MDD subjects with anhedonia [5]. Furthermore, even if this symptomatic response to antidepressant treatment is present, the persistence of anhedonia complaints may favor the maintenance of alterations in psychosocial functioning in MDD subjects [6]. Moreover, at the somatic level, MDD subjects with anhedonia seem to be more at risk of developing cardiometabolic diseases than MDD subjects without anhedonia [7]. However, despite this central place of anhedonia and its potential negative consequences, this symptom is not systematically present in all MDD subjects since the DSM 5 criteria may allow a diagnosis of MDD in the absence of anhedonia [8]. In this context, it therefore seems necessary to identify the factors favoring changes in anhedonia complaints in MDD subjects in order to allow better prevention of the potential negative consequences associated with the occurrence of this depressive symptom in this particular subpopulation.

In the literature, there seems to be a particular relationship between anhedonia and low-grade inflammation defined by C-Reactive Protein (CRP) levels >3 mg/L but <10 mg/L [9]. Indeed, low-grade inflammation seems to induce alterations in neurotransmission and neuronal circuits involved in the reward process, favoring the occurrence of anhedonia complaints in multiple psychiatric disorders [10]. However, despite a high prevalence of low-grade inflammation in MDD subjects, few studies have investigated the impact of this low-grade inflammation on changes in anhedonia complaints in this particular subpopulation [11,12,13,14,15]. Furthermore, in most currently available studies, the samples of MDD subjects recruited were generally small, which may limit the interpretation of their results [11,13,14,15]. Based on these elements, it could therefore be interesting to investigate the potential role played by low-grade inflammation in recent changes in anhedonia (defined as the recent onset or worsening of this symptom within the past 2 weeks) among MDD subjects to allow a better understanding of the pathophysiology of this depressive symptom in this subpopulation.

Thus, this study aimed to investigate the potential role of low-grade inflammation in the recent change in anhedonia among MDD subjects. The hypothesis of this study was that low-grade inflammation may act as a risk factor in MDD, potentially exacerbating symptoms and contributing to the recent onset or worsening of anhedonia complaints within the past 2 weeks. The objective of this approach was to provide health professionals with reliable data on the potential role of low-grade inflammation in the recent change in anhedonia complaints in MDD subjects, thereby opening new perspectives for the understanding and, subsequently, the management of this symptom in this particular subpopulation.

## 2. Materials and Methods

### 2.1. Population

The 496 MDD subjects were retrospectively recruited from the clinical database of the Erasme Hospital Sleep Laboratory for the period from 1 January 2017 to 31 December 2023. Prior to their hospitalization at the Sleep Unit for polysomnography, all these MDD subjects had benefited from an assessment by physicians specialized in sleep medicine during a specific outpatient care pathway. These polysomnographic recordings had been carried out on these MDD subjects to allow an objective assessment of their sleep complaints and exclude the presence of comorbid sleep disorders negatively impacting mood regulation. Finally, in this study, we did not recruit individuals without MDD because our objective was to focus on the subpopulation of MDD subjects where the occurrence of low-grade inflammation may potentially promote the onset or worsening of some depressive symptoms.

The inclusion criteria were age ≥ 18 years and the presence of a major depressive episode meeting DSM 5 criteria [8].

The exclusion criteria were as follows: the presence of any psychiatric disorder other than MDD, severe uncontrolled somatic conditions (such as chronic hepatic disease, chronic pancreatic disease, severe cardiovascular disease, chronic pulmonary disease, severe renal disease, autoimmune diseases, and conditions affecting the hypothalamic–pituitary–adrenal axis like Cushing’s syndrome), infectious or inflammatory diseases, CRP levels greater than 10 mg/L, pregnancy, central hypersomnia (including narcolepsy or primary hypersomnia), predominantly central sleep apnea syndrome, known or treated obstructive sleep apnea syndrome prior to the sleep laboratory assessment, current or past head trauma, current or past central nervous system lesions impacting respiratory centers, craniofacial or rib cage malformations, and current or past substance use disorders.

### 2.2. Medical and Psychiatric Assessment

Each recruited MDD subject had a medical interview and a comprehensive somatic assessment during their hospital admission process. This assessment included physical examination, fasting blood tests, electrocardiograms, daytime electroencephalograms, and urinalysis to diagnose any potential somatic comorbidities.

The CRP levels were only assessed once using immuno-turbidimetry on plasma during the admission assessment. The material was a Roche CRP4-Cobas with a minimum detection level of 0.3 mg/L. Low-grade inflammation was defined as absent when CRP levels were ≤3 mg/L and as present when CRP levels were >3 mg/L (and <10mg/L) [16,17].

A psychiatric evaluation was performed by a psychiatrist from the unit on all MDD subjects to diagnose their potential psychiatric comorbidities according to the above-mentioned DSM diagnostic criteria. Afterwards, all these subjects completed a series of a self-questionnaires assessing depressive symptoms (21-items Beck Depression Inventory [BDI-II]), anxiety symptoms (Spielberger trait and state questionnaire), insomnia complaints (Insomnia Severity Index) and daytime sleepiness (Epworth Sleepiness Scale) [18,19,20,21] (detailed description in Supplementary Data—S1).

Recent change in anhedonia was assessed by the Anhedonia Subscale of BDI-II [22]. Joiner et al. (2003) [22] proposed assessing anhedonia based on three specific items from the BDI-II, targeting the loss of pleasure or interest in the following: usual activities or field of interest, interactions with other people, and sex. Each item describes a decreasing range of interest, from no change to complete disinterest. However, given the absence of validated cut-offs for this BDI Anhedonia subscale, recent change in anhedonia was considered present when the score was >3, which seemed to be most consistent with the psychometric properties of this subscale demonstrated in the article by Joiner et al. (2003) [22]. Thus, the recent change in anhedonia corresponds to the recent onset or worsening of this symptom within the past 2 weeks.

Finally, based on a sleep-specific anamnesis and the results of the polysomnography (Supplementary Data—S2), a diagnosis of potential sleep comorbidities (sleep apnea syndrome, insomnia disorder, and nocturnal movement disorder) was made by the physicians assigned to the Sleep Unit for all MDD subjects included in this study.

### 2.3. Statistics

Statistical analyses were performed using Stata software version 14. The anormal distribution of the data was verified through histograms, scatter plots, and quantile–quantile plots, while the equality of variance was assessed using Leven’s test.

To facilitate our analyses, we divided our sample of MDD subjects into two groups: a control group without recent change in anhedonia (Anhedonia subscale of the BDI-II ≤ 3) and a patient group with recent change in anhedonia (Anhedonia subscale of the BDI-II > 3).

Categorical data were described by percentages and numbers, while continuous variables were described according to their distribution by their median and P25-P75. The asymmetrically and dichotomously distributed data were analyzed with Wilcoxon and Chi^2^ tests, respectively.

Univariate logistic regression models were used to investigate the risk for recent change in anhedonia associated with low-grade inflammation and potential confounding factors. Following a review of the literature on factors associated with anhedonia [23,24,25,26,27,28,29,30,31,32,33,34], potential confounding factors included in this study were age (categorized as <50 years, ≥50 years), body mass index (categorized as <25 kg/m^2^, ≥25 and <30 kg/m^2^, ≥30 kg/m^2^), obstructive sleep apnea syndrome (categorized as absent, mild, moderate to severe), sleep movement disorders (categorized as absent, moderate to severe periodic limb movement syndrome alone, and restless legs syndrome alone or combined with periodic limb movements), Epworth Sleepiness Scale score (categorized as <16, ≥16), anxiety symptoms (categorized as absent, trait anxiety alone, state anxiety alone, trait and state anxiety), depression severity (BDI reduced to 18 items) (categorized as score <15, score ≥15), MDD duration (categorized as <6 months, ≥6 months) and binary variables: gender, antidepressant therapy, benzodiazepine receptor agonists, other psychotropic medications, smoking, alcohol consumption, caffeine consumption, hypertension, dyslipidemia, type 2 diabetes, cardiovascular comorbidities, and insomnia disorders.

Since the population recruited retrospectively for this study consisted solely of MDD subjects referred to the sleep unit for polysomnography following sleep complaints, it was decided to include potential comorbid sleep disorders and complaints of excessive daytime sleepiness among these potential confounding factors. Indeed, the literature has shown that both sleep disorders and alterations in daytime functioning are associated with higher incidence of anhedonia complaints in MDD patients [33,35,36,37,38]. Furthermore, given the data available in the literature, there seems to be evidence to support a central role of anxiety symptoms in the occurrence of anhedonia complaints in MDD subjects [34,39], which justifies taking these symptoms into account among the potential confounding factors investigated in this study. In this context, the results of the different scales (Spielberger trait and state questionnaire, Insomnia Severity Index, and Epworth Sleepiness Scale) used during the standardized assessment of all subjects admitted to the sleep unit were extracted from the database to allow these analyses.

In multivariate logistic regression models, the risk for recent change in anhedonia associated with low-grade inflammation was only adjusted for significant confounders in univariate analyses. Since the objective of this study was to focus solely on the potential role played by low-grade inflammation in the occurrence of a recent change in anhedonia in MDD subjects, the aim of this approach already used in previous studies for multivariate analyses [40,41,42,43] was to allow systematic consideration of the impact of these potential confounding factors highlighted during our univariate analyses in order to allow the best possible interpretation of the results obtained.

Following the conditions of use of multivariate logistic regression analyses (number of subjects per cofactor ≥ 10), each of the two groups of patients for this study had to contain at least 190 subjects (10 subjects * 19 cofactors) to ensure the validity of the analyses performed, which was achieved in this study.

The adequacy of the final models was assessed using the Hosmer–Lemeshow test, while the specification of the model was evaluated using the Link test.

Results were considered significant when the *p*-value was <0.05.

## 3. Results

### 3.1. Descriptive and Univariate Analyses (Table 1 and Table 2)

In our cohort, the MDD group with recent change in anhedonia is associated with gender, age, treatment (antidepressant and psychotropic drugs other than benzodiazepine), MDD duration, type 2 diabetes and sleep disturbances (presence of insomnia disorder and Epworth Sleepiness Scale score ≥16). The prevalence of low-grade inflammation was 30.2% in the MDD population and higher in the MDD group with recent change in anhedonia (37.7%) compared to the MDD group without recent change in anhedonia (25.2%). Finally, recent change in anhedonia was present in 40.1% of MDD subjects and is associated with elevated other symptoms including depression, anxiety, and insomnia.

**Table 1:** Descriptive analyses (n = 496).

Variables	Categories	%	Subjects Without Recent Change in Anhedonia	Subjects with Recent Change in Anhedonia	*p*-Value Chi2	Cramer’s V
Gender	Female (n = 281)	56.7%	52.9%	62.3%	0.037	0.094
	Male (n = 215)	43.3%	47.1%	37.7%		
Age (years)	<50 (n = 330)	66.5%	70.0%	61.3%	0.044	0.091
	≥50 (n = 166)	33.5%	30.0%	38.7%		
BMI (kg/m^2^)	<25 (n = 145)	29.2%	31.0%	26.6%	0.380	0.062
	≥25 and <30 (n = 164)	33.1%	33.7%	32.2%		
	≥30 (n = 187)	37.7%	35.3%	41.2%		
Antidepressant therapy	No (n=352)	71.0%	80.5%	56.8%	<0.001	0.256
	Yes (n=144)	29.0%	19.5%	43.2%		
Benzodiazepine receptor agonists	No (n = 427)	86.1%	88.2%	82.9%	0.095	0.075
	Yes (n = 69)	13.9%	11.8%	17.1%		
Other psychotropic medications	No (n = 391)	78.8%	84.9%	69.9%	<0.001	0.180
	Yes (n = 105)	21.2%	15.1%	30.1%		
Smoking	No (n = 405)	81.7%	82.8%	79.9%	0.409	0.037
	Yes (n = 91)	18.3%	17.2%	20.1%		
Alcohol	No (n = 268)	54.0%	50.8%	58.8%	0.082	0.078
	Yes (n = 228)	46.0%	49.2%	41.2%		
Caffeine	No (n = 72)	14.5%	16.5%	11.6%	0.126	0.069
	Yes (n = 424)	85.5%	83.5%	88.4%		
Type 2 diabetes	No (n = 420)	84.7%	89.9%	76.9%	<0.001	0.177
	Yes (n = 76)	15.3%	10.1%	23.1%		
Dyslipidemia	No (n = 263)	53.0%	54.6%	50.8%	0.407	0.037
	Yes (n = 233)	47.0%	45.4%	49.2%		
Hypertension	No (n = 308)	62.1%	64.0%	59.3%	0.293	0.047
	Yes (n = 188)	37.9%	36.0%	40.7%		
Cardiovascular comorbidities	No (n = 449)	90.5%	92.3%	87.9%	0.108	0.072
	Yes (n = 47)	9.5%	7.7%	12.1%		
OSAS	No (n = 209)	42.1%	42.8%	41.2%	0.404	0.061
	Mild (n = 126)	25.4%	26.9%	23.1%		
	Moderate to severe (n = 161)	32.5%	30.3%	35.7%		
Insomnia disorder	No (n = 136)	27.4%	31.0%	22.1%	0.030	0.097
	Yes (n = 360)	72.6%	69.0%	77.9%		
Sleep movement disorders	No (n = 414)	83.5%	82.8%	84.4%	0.788	0.031
	Moderate to severe PLMs alone (n = 32)	6.5%	7.1%	5.5%		
	RLS alone or combined with PLMs (n = 50)	10.0%	10.1%	10.1%		
ESS	<16 (n = 403)	81.3%	85.2%	75.4%	0.006	0.123
	≥16 (n = 93)	18.7%	14.8%	24.6%		
Anxiety	No (n = 198)	39.9%	47.8%	28.1%	<0.001	0.297
	Trait anxiety alone (n = 56)	11.3%	9.8%	13.6%		
	State anxiety alone (n = 81)	16.3%	20.2%	10.6%		
	Trait + state anxiety (n = 161)	32.5%	22.2%	47.7%		
BDI reduced to 18 items	<15 (n = 173)	34.9%	44.1%	21.1%	<0.001	0.237
	≥15 (n = 323)	65.1%	55.9%	78.9%		
MDD duration (months)	<6 (n = 308)	62.1%	70.4%	49.8%	<0.001	0.208
	≥6 (n = 188)	37.9%	29.6%	50.2%		
LGI	No (n = 346)	69.8%	74.8%	62.3%	0.003	0.133
	Yes (n = 150)	30.2%	25.2%	37.7%		
Recent change in anhedonia	No (n = 297)	59.9%				
	Yes (n = 199)	40.1%				
	Median (P25–P75)				Wilcoxon test	Effect size r
Age (years)	43 (33–52)		42 (32–51)	45 (34–53)	0.040	−0.092
BMI (kg/m^2^)	27.9 (24.6–33.2)		27.4 (24.2–32.6)	28.7 (24.8–33.5)	0.110	−0.072
ESS	11 (7–14)		10 (8–14)	11 (6–15)	0.562	−0.003
ISI	17 (14–20)		17 (13–20)	18 (15–21)	0.015	−0.110
Spielberger Trait Anxiety Inventory	58 (51–65)		55 (50–61)	62 (56–69)	<0.001	−0.355
Spielberger State Anxiety Inventory	59 (51–68)		57 (50–65)	62 (52–74)	<0.001	−0.209
BDI	19 (16–27)		17 (14–21)	27 (20–35)	<0.001	−0.564
BDI reduced to 18 items	17 (13–23)		15 (12–19)	22 (15–29)	<0.001	−0.405
BDI–anhedonia	3 (2–4)		2 (1–3)	5 (4–6)	<0.001	−0.858

BMI = body mass index, OSAS = obstructive sleep apnea syndrome, PLMs = periodic limb movements during sleep, RLS = restless legs syndrome, LGI = low-grade inflammation, ESS = Epworth sleepiness scale, ISI = insomnia severity index, BDI = Beck depression inventory, MDD = major depressive disorder.

**Table 2:** Univariate logistic regression models to predict the risk for recent change in anhedonia associated with low-grade inflammation and potential confounding factors (n = 496).

Variables	Categories	OR (CI 95%)	*p*-Value
Gender	Female	1	0.038
	Male	0.68 (0.47 to 0.98)	
Age (years)	<50	1	0.044
	≥50	1.48 (1.01 to 2.15)	
BMI (kg/m^2^)	<25	1	0.381
	≥25 and <30	1.11 (0.70 to 1.76)	
	≥30	1.36 (0.87 to 2.11)	
Antidepressant therapy	No	1	<0.001
	Yes	3.14 (2.10 to 4.68)	
Benzodiazepine receptor agonists	No	1	0.096
	Yes	1.54 (0.93 to 2.57)	
Other psychotropic medications	No	1	<0.001
	Yes	2.42 (1.56 to 3.75)	
Smoking	No	1	0.409
	Yes	1.21 (0.77 to 1.92)	
Alcohol	No	1	0.082
	Yes	0.72 (0.50 to 1.04)	
Caffeine	No	1	0.128
	Yes	1.51 (0.89 to 2.57)	
Type 2 diabetes	No	1	<0.001
	Yes	2.68 (1.62 to 4.42)	
Dyslipidemia	No	1	0.407
	Yes	1.16 (0.81 to 1.67)	
Hypertension	No	1	0.293
	Yes	1.22 (0.84 to 1.76)	
Cardiovascular comorbidities	No	1	0.110
	Yes	1.63 (0.89 to 2.98)	
OSAS	No	1	0.405
	Mild	0.89 (0.56 to 1.41)	
	Moderate to severe	1.22 (0.81 to 1.85)	
Insomnia disorder	No	1	0.031
	Yes	1.58 (1.04 to 2.40)	
Sleep movement disorders	No	1	0.789
	Moderate to severe PLMs alone	0.77 (0.36 to 1.63)	
	RLS alone or combined with PLMs	0.98 (0.54 to 1.78)	
ESS	<16	1	0.007
	≥16	1.88 (1.19 to 2.96)	
Anxiety	No	1	<0.001
	Trait anxiety alone	2.36 (1.28 to 4.34)	
	State anxiety alone	0.89 (0.49 to 1.59)	
	Trait + state anxiety	3.65 (2.35 to 5.67)	
BDI reduced to 18 items	<15	1	<0.001
	≥15	2.95 (1.96 to 4.45)	
MDD duration (months)	<6 (n = 308)	1	<0.001
	≥6 (n = 188)	2.40 (1.65 to 3.48)	
LGI	No	1	0.003
	Yes	1.79 (1.21 to 2.64)	

BMI = body mass index, OSAS = obstructive sleep apnea syndrome, PLMs = periodic limb movements during sleep, RLS = restless legs syndrome, LGI = low-grade inflammation, ESS = Epworth sleepiness scale, BDI = Beck depression inventory, MDD = major depressive disorder.

### 3.2. Multivariate Analyses (Table 3)

After adjusting for main confounders identified during univariate analyses, multivariate logistic regression analyses showed that low-grade inflammation was associated with recent change in anhedonia in MDD subjects (OR 1.77, CI 95% [1.14 to 2.75], *p* = 0.012). Detailed analyses for each multivariate model tested are available in Supplementary Data—S3.

**Table 3:** Multivariate logistic regression models to predict the risk for recent change in anhedonia associated with low-grade inflammation after adjustment for confounding factors (n = 496).

Variable	Model	Category	OR	Category	OR	*p*-Value
Low-grade Inflammation	1	No	1.00	Yes	1.73 (1.15–2.62)	0.009
	2		1.00		1.65 (1.08–2.50)	0.019
	3		1.00		1.71 (1.12–2.61)	0.013
	4		1.00		1.77 (1.14–2.75)	0.012

Model 1 = Model adjusted for gender, age, antidepressant therapy and other psychotropic medications. Model 2 = Model adjusted for gender, age, antidepressant therapy, other psychotropic medications and type 2 diabetes. Model 3 = Model adjusted for gender, age, antidepressant therapy, other psychotropic medications, type 2 diabetes, insomnia disorder and ESS scores. Model 4 = Model adjusted for gender, age, antidepressant therapy, other psychotropic medications, type 2 diabetes, insomnia disorder, ESS scores, anxiety symptoms, depression severity and MDD duration. ESS = Epworth sleepiness scale, MDD = major depressive disorder.

### 3.3. Polysomnographic Results (Table 4)

The analysis revealed that subject with recent change in anhedonia exhibited reduced total sleep time, which was associated with lower sleep efficiency, a decreased percentage of rapid eye movement (REM), and an increased REM latency. In addition, the percentage of wakefulness after sleep onset was higher in the MDD group with recent change in anhedonia.

**Table 4:** Polysomnographic data (n = 496).

	Whole Sample	Subjects Without Recent Change in Anhedonia	Subjects with Recent Change in Anhedonia	*p*-Value
Sleep latency (min)	62.5 (33.8–109.3)	59.5 (31.0–105.5)	72.5 (37.0–114.0)	0.175
Sleep efficiency (%)	73.2 (61.9–82.0)	74.6 (64.5–82.4)	71.1 (58.0–81.7)	0.021
Sleep period time (min)	438.5 (400.0–472.3)	441.5 (405.0–474.5)	434.0 (391.5–468.0)	0.177
Total sleep time (min)	379.3 (329.0–421.3)	387.5 (339.0–424.5)	372.5 (304.5–412.5)	0.013
% stage 1	7.0 (4.7–9.9)	7.3 (5.2–9.9)	6.9 (4.3–9.8)	0.151
% stage 2	50.3 (42.3–57.0)	50.1 (41.9–57.0)	50.3 (42.8–57.2)	0.760
% Stage 3	12.2 (5.3–18.0)	12.8 (5.4–19.0)	11.0 (5.1–17.1)	0.280
% REM sleep	15.9 (10.8–20.3)	16.3 (11.6–20.5)	14.8 (9.2–19.5)	0.020
REM latency (min)	92.0 (67.5–155.0)	87.8 (64.5–136.0)	100.5 (72.5–186.5)	<0.001
% wake after sleep onset	11.1 (5.7–19.3)	10.0 (5.4–18.5)	12.1 (6.2–21.0)	0.049
Number of awakenings	24 (17–33)	23 (17–32)	24 (17–34)	0.654
Micro-arousal index	11 (6–17)	11 (7–16)	10 (6–18)	0.725
Apnea–hypopnea index	7 (2–19)	7 (2–18)	8 (2–21)	0.227
Oxygen desaturation index	4 (1–13)	4 (1–12)	4 (1–15)	0.248
Total time under 90% of SaO_2_ (min)	0.5 (0.0–9.0)	0.0 (0.0–8.5)	0.5 (0.0–12.0)	0.281
PLMS index	1 (0–7)	2 (0–8)	1 (0–7)	0.211
	Median (P25–P75)	Median (P25–P75)	Median (P25–P75)	Wilcoxon test

REM = rapid eye movement, SaO_2_ = oxygen saturation, PLMS = periodic limb movements during sleep.

## 4. Discussion

### 4.1. Population Characteristics and Prevalence

In our cohort, MDD subjects who reported recent change in anhedonia were predominantly female, over the age of 50, and exhibited a higher prevalence of type 2 diabetes compared to those without recent change in this symptom. This group was also more frequently treated with selective serotonin reuptake inhibitors (SSRIs)/serotonin and norepinephrine reuptake inhibitors (SNRIs) and other psychotropic medications than benzodiazepine. Despite receiving more pharmacological treatment, patients in this group also displayed more severe clinical symptoms, such as anxiety, depressed mood, and sleep disturbances. Polysomnography findings further indicated that MDD subjects with recent change in anhedonia demonstrated poorer sleep quality, reduced REM sleep, prolonged REM latency, and increased percentage of wakefulness after sleep onset. However, the higher percentage of treated subjects—as previously noted—may interfere with the interpretation of these results. The prevalence of recent change in anhedonia among MDD subjects in our cohort (40.1%) aligns with existing literature, which reports rates ranging from 37% to 72%, depending on the scale used and the population studied [2,44,45,46]. While there is a well-established consensus regarding the higher prevalence of MDD among women, similar gender difference in anhedonia as we found in our result showed mixed results in existing studies [46]. Our cohort includes subjects with sleep complaints; therefore, the higher prevalence of sleep disturbances in women could explain this result [47,48,49].

### 4.2. Inflammation

The prevalence of low-grade inflammation in the MDD sample is consistent with previous studies [17]. Moreover, the MDD group with recent change in anhedonia displayed elevated levels of low-grade inflammation, in line with our research hypothesis. This association remained after adjusting for the main confounding variables identified in the univariate analysis. MDD is associated with inflammation which could constitute a deleterious context underlaying the psychiatric pathology. The main hypothesis linking depression and inflammation is the concept of Sickness Behavior [50,51]. During an inflammatory episode, cytokines could cross the blood–brain barrier and trigger neurovegetative, somatic, and psychological symptoms, including depressed mood, anxiety, and anhedonia [52]. In contrast to an infectious episode, where the symptoms stop as the disease does, persistent cytokine activation in the case of depression, associated with chronic inflammation, could continue to sustain psychological symptoms, thereby contributing to enhance MDD. The inflammatory marker of low-grade inflammation is the levels of CRP. CRP is a protein released by the liver in response to the proinflammatory cytokine IL-6. Notably, IL-6 has been specifically associated with anhedonia and with striatum atrophy [53]. Consistently, J Savitz et al. (2025) have shown that MDD subjects with low-grade inflammation showed elevated IL-6 levels and anhedonia symptoms in case of inflammatory challenge [54].

From a neurobiological perspective, anhedonia is associated with a deficit in reward processing. The reward system encompasses perception of pleasure but also coding of reward value, assessing costs and benefits, learning from prior reinforcement, evaluating effort, and making decisions that lead to action [55]. In the DSM-5, anhedonia is described as a deficit in hedonic experience of rewards and motivation for rewards [8]. However, anhedonia has been lately conceptualized and validated by several studies as a deficit across three partially separable subtypes of reward processing: reward liking, reward wanting, and reward learning [56,57,58,59]. Reward liking corresponds to the consuming phase reliant on the pleasure of rewards. Reward wanting refers to the anticipatory phase and incentive motivation based on motivation driving individuals towards rewards. Finally, reward learning refers to learning from outcomes to adjust the accuracy of future decisions [57,60]. The neurobiological feature of anhedonia has been extensively assessed with neuroimaging (i.e., functional magnetic resonance imaging) and showed common frontostriatal abnormalities across the three parts. Frontostriatal abnormalities refer to disruptions in the functional and structural connections between the frontal cortex and the striatum; both regions are involved with cognitive and behavioral functions. Evidence showed that the neural pattern could be partially dissociated for each subtype due to hyper- or hypoactivation of the different brain areas and different neurotransmitters involved. Likewise, in reward liking and wanting, striatal hypoactivation has been observed, at the same time as hypoactivation and hyperactivation across dissociable frontal regions. For reward learning, it has been shown that the sensitivity of the frontostriatal network to positive feedback was decreased without neural abnormalities for negative feedback. Furthermore, striatal hypoactivation observed in the reward liking could be associated with abnormal opioid signaling, while those observed in the reward wanting could be associated with dopamine signaling. The neural abnormalities observed in reward learning could also be associated with the alteration in dopamine signaling. Likewise, acute enhancement of dopaminergic transmission has been demonstrated to potentiate reward-related striatal activation and corticostriatal functional connectivity in individuals suffering from depression [56]. Thus, these subtypes could have partially different neural bases (i.e., area and/or signaling). Borsini et al. (2020) have suggested that individuals suffering from this symptom could present different combinations of these dissociable neural subtypes leading to particular phenotypes of anhedonia, thereby paving the way for interindividual differences and treatments [61]. Inflammation also seems to induce alterations in neurotransmission and neuronal circuits involved in the reward process favoring the occurrence of anhedonia complaints in multiple psychiatric disorders [10,11]. It has been shown that inflammation “per se” could trigger similar frontostrial abnormalities [62]. Therefore, anhedonia (as aforementioned in our results) is associated with inflammation that could contribute to modulating neural circuits [63]. Notably, Felger et al. (2016) have shown, for MDD, a positive association between the CRP levels, anhedonia symptoms, and the decreased functional connectivity within the corticostriatal reward system [64].

### 4.3. Perspectives Opened by the Results of This Study for the Management of MDD Subjects with Anhedonia and Low-Grade Inflammation

In this study, most MDD subjects receiving antidepressant treatment were taking SSRIs or SNRIs. However, it has been shown that despite a potential effect of these antidepressant treatments on anhedonia complaints, these molecules are not the most effective against this depressive symptom [65]. Furthermore, there is evidence to suggest increased resistance to SSRIs and SNRIs in the presence of low-grade inflammation in MDD subjects [66]. In this context, the results of our study seem to indicate that it could be interesting to identify new therapeutic strategies in anhedonic MDD subjects with low-grade inflammation in order to allow better management of this specific subgroup of patients.

#### 4.3.1. Conventional Treatment

While there is growing consensus on the link between inflammation and MDD, the clinical application, whether in diagnosis or treatment, remains unclear. Furthermore, there is no clear agreement on which specific symptoms of MDD—and to what extent—may be responsive to anti-inflammatory interventions. Thus, it has been shown that inflammation could interfere with the effectiveness of the antidepressant and that SSRIs, the first line treatment for depression could be less effective in case of anhedonia [46]. Likewise, Buproprion is a combined treatment for MDD that targets both dopaminergic and serotoninergic circuits compared to SSRI drugs which act only on the serotoninergic system. In the context of depression associated with inflammation, Buproprion has shown better treatment response than SSRI alone [67]. Moreover, Tomarken et al. (2004) have shown that the dopaminergic effect of Bupropion could be effective in the treatment of anhedonia symptoms [68].

#### 4.3.2. Other Treatments

MDD is a multifactorial psychiatric disorder that necessitates a multidisciplinary approach to recovering. Notably, as discussed before, the neurobiological basis of anhedonia overlaps with patterns of hypo- or hyperactivation in brain regions associated with inflammation, suggesting that anhedonia could be a responding symptom in cases of low-grade inflammation.

The endocannabinoids are endogenous lipid-based signaling molecules. They exert their effects by binding to cannabinoid receptors, which are widely expressed throughout the brain (CB1-receptor in particular). These molecules are key components of the endocannabinoid system, a complex network comprising cannabinoid receptors, endocannabinoids, and the enzymes that regulate their biosynthesis and degradation. The endocannabinoid system plays an important role in the central nervous system and may also exhibit anti-inflammatory properties [69,70,71]. Diet could act as a key factor of synthesis and release of endocannabinoids and other related mediators through the n-3 polyunsaturated fatty acid (omega 3) [72,73]. Omega 3 have anti-inflammatory properties and could modulate microbiota-derived endocannabinoid-like mediators as well [72,73]. In addition, Minichino et al. (2021) have shown that the diversity of the gut microbiota could modulate anhedonia through the endocannabinoid system [74]. The Mediterranean diet which, first and foremost, enhances an abundant and healthy gut microbiota also has anti-inflammatory properties “per se” that could act in this context [75,76]. In contrast, it has been shown that subjects suffering from anhedonia consume more high-glycemic foods which could foster diet-related pathologies such as Type 2 Diabetes as in MDD [77]. High-glycemic foods are able to dramatically increase glycemia (in a physiological range) and are associated with oxidative stress and inflammation [78,79]. In addition, it has been shown in animal models that a high-sugar, high-fat diet could be associated with anhedonia, potentially involving the receptor for advanced glycation end products (RAGE) as a mediating factor [80]. The AGE(s) are formed through the no-enzymatic reaction between reduced sugars (with free carbonyls groups) and protein free amino groups, lipids, or nucleic acids [81]. AGE(s) are a heterogeneous compound family and normal byproducts of the metabolism but could increase in the case of oxidative stress. AGE(s) could also come from endogenous sources such as diet. A diet rich in sugar, fat, and meat with a high-temperature cooking process could increase the AGE content [82]. The AGE receptor (RAGE) is an extracellular multiligand receptor expressed in epithelial cells, cardiomyocytes, macrophages, adipocytes, microglia, astrocytes, and neurons. The ligand–receptor binding (AGE_RAGE) triggers intracellular proinflammatory pathways such as nuclear factor-kappa B (NF-kB) and mitogen-activated (MAP) protein leading to the release of oxygen species (ROS) and proinflammatory cytokines, subsequently enhancing inflammation [82]. Furthermore, studies have shown that chronic stress induces anhedonia in wild-type mice, in contrast to RAGE knockout mice under the same conditions [83]. Notably, anxiety is associated with oxidative stress and inflammation “per se”. Thus, excessive AGE levels increased by oxidative stress due to anxiety or unhealthy diet could contribute to sustaining inflammation and anhedonia [80].

### 4.4. Limitations

Although previous studies have already used samples of MDD subjects from sleep units [2,16,84], it cannot be excluded that this specific recruitment is associated with the existence of a selection bias, which may limit the generalizability of our results. However, in order to minimize, as much as possible, this potential risk of bias induced by an overrepresentation of comorbid sleep disorders in our sample, the results of this study were adjusted for sleep disorders significantly associated with recent change in anhedonia in univariate analyses. Moreover, since the outcome of this study was only the recent change in anhedonia (assessed through specific items of the BDI-II), it might be interesting to confirm the results obtained using a dedicated anhedonia scale (such as the Snaith-Hamilton Pleasure Scale [85]) in order to complement them with a measure of anhedonia not limited to recent change alone, which would allow for a more global understanding of the potential role played by low-grade inflammation in the pathophysiology of anhedonia complaints both in terms of their occurrence (anhedonia state) and their modification (recent change in anhedonia) in MDD subjects. Finally, the results obtained in this study come from retrospective data that, even if they have been encoded in a systematic manner, cannot be verified directly with these MDD subjects referred for polysomnography, which means that these findings need to be replicated in prospective and longitudinal studies with a recruitment of MDD subjects not only limited to populations from sleep units.

## 5. Conclusions

While the relationship between inflammation and depression is now well-established, its implications for the physiopathology and treatment of MDD remain to be clarified. Anhedonia (especially its recent change), a core symptom of MDD, appears to be particularly linked to inflammation, as confirmed by our results and existing neurobiological evidence. Moreover, targeting anhedonia within the context of inflammation and its underlying neurobiological mechanisms could potentially offer a promising approach for advancing our understanding of the physiopathology of MDD and developing more effective treatments to improve patient prognosis. In this context, since some elements available in the literature seem to indicate that the inflammatory process would be counteracted by various strategies—either individually or in combination (such as pharmacological treatment and/or dietary interventions)—it is essential to continue research on therapeutic approaches associated with a potential reduction in this low-grade inflammation in MDD subjects in order to allow for more adequate management of anhedonia complaints in this particular subpopulation.

## Data Availability

The data presented in this study are available on request from the corresponding author (the data are not publicly available due to privacy restrictions).

## Supplementary Materials

The following supporting information can be downloaded at: https://www.mdpi.com/article/10.3390/medicina61122125/s1, S1: Detailed description of the questionnaires used; S2: Specific care pathway for sleep assessment; S3: Detailed analyses for each multivariate model tested. References [86,87,88,89,90,91,92,93] are cited in Supplementary Materials.
