# Supplementary material for: Recent Change in Anhedonia, Major Depression and Low-Grade Inflammation: Dangerous Liaisons? A Study Based on a Cohort Referred for Polysomnography
Source: Medicina (Kaunas). 2025 Nov 28;61(12):2125. doi: 10.3390/medicina61122125 (PMC12735016; doi:10.3390/medicina61122125)
Supplement: Supplementary file 1 [file medicina-61-02125-s001.zip › medicina-3874457-supplementary.pdf]

Article

Anhedonia, Major depression and Low-grade inflammation: dangerous liaisons? A cohort study

S1/Detailed description of the questionnaires used

- Depressive symptom was measured with the 21-items Beck Depression Inventory (BDI-II). Each item may be scored from 0 to 3, which means that the total score may vary from 0 to 63. Depressive symptoms were considered absent if the score was 0 to 9, mild if the score was 10 to 18, moderate if the score was 19 to 29, and severe if the score was over 30.

- The Spielberger questionnaire was used to investigate the presence of anxiety symptoms. The state section assesses anxiety at the time of polysomnographic recording, while the trait section assesses anxiety in daily life. These two sections each comprise 20 questions scored from 1 to 4. For each section, the score can vary from 20 to 80. The lower the final score, the lower the level of anxiety, and vice versa.

- The Insomnia Severity Index was used to assess symptoms of insomnia. The 7 items on this index score from 0 to 4, resulting in a total score that can range from 0 to 28. Insomnia symptoms are categorized as follows: absent for scores between 0 and 7, subclinical for scores between 8 and 14, moderate for scores between 15 and 21, and severe for scores of 22 and above.

- The Epworth Sleepiness Scale was used to evaluate excessive daytime sleepiness. The scale comprises 8 items which assess daytime sleepiness in different daytime situations. Each item may score from 0 to 3, leading to a total that can range from 0 to 24. A score exceeding 10 is indicative of excessive daytime sleepiness.

S2/Specific care pathway for sleep assessment

S.2.1/Sleep assessment and examination

Sleep history: The sleep laboratory psychiatrist conduct an interview for all individuals admitted to perform a complete inventory of their sleep-related complaints, including sleep habits, severity of self-reported insomnia complaints (difficulty falling asleep, repeated nocturnal awakenings, early morning awakening and unrefreshing sleep), symptoms related to sleep apneas (snoring and self-reported apneas), symptoms related to restless legs syndrome (impatience of the legs with or without abnormal sensations: aggravated by rest, partially or temporarily relieved by movement and increased during the evening or night) and abnormal nocturnal movements (periodic limb movements).

S.2.2/Polysomnographic recording

Subjects with major depressive disorder (MDD) underwent polysomnographic recording, which included the following parameters: sleep latency, sleep efficiency, total sleep period, total sleep time (stages 1, 2, 3), percentage of rapid eye movement (REM) sleep and REM latency, percentage of wake after sleep onset, number of awakenings, microarousal index, apnea-hypopnea index, oxygen desaturation index, total time with oxygen saturation below 90% (minutes), and periodic limb movements during sleep (PLMS). The polysomnography was conducted in accordance with the recommendations of the American Academy of Sleep Medicine (Kushida et al., 2005).

Patients go to bed between 10:00 PM and midnight and wake up between 6:00 AM and 8:00 AM, according to their usual routine. During bedtime, patients must lie in bed with the lights off. Daytime naps are not allowed.

The polysomnographic setup used included: two electro-oculogram channels, three electroencephalogram channels, one submental electromyogram channel, one electrocardiogram channel, and thermistors for detecting oronasal airflow, one microphone to record respiratory sounds and snoring. Piezoelectric sensors and strain gauges were also used to measure thoracic and abdominal respiration, along with electrodes to monitor leg movements. Polysomnographic recordings are visually scored by trained technicians in accordance with the criteria established by the American Academy of Sleep Medicine (“AASM Scoring Manual - American Academy of Sleep Medicine,” n.d.). Apneas are scored when there is a ≥90% reduction in airflow lasting at least 10 seconds, while hypopneas are scored when there is a ≥30% reduction in airflow for at least 10 seconds, accompanied by a ≥3% drop in oxygen saturation or followed by a microarousal (Berry et al., n.d.). The apnea–hypopnea index (AHI) is defined as the total number of apneas and hypopneas divided by the total sleep time in hours. Obstructive Sleep Apnea (OSA) is considered absent when the apnea-hypopnea index is <5/hour, mild when the apnea-hypopnea index is ≥5/hour and <15/hour and moderate to severe when the apnea-hypopnea index is ≥15/hour (Fleetham et al., 2006). Periodic limb movements are scored based on the following criteria:: 1) a duration between 0.5 and 10 seconds, 2) an interval between 5 and 90 seconds from the onset of limb movement, and 3) the movements are part of a sequence of at least 4 consecutive movements meeting the preceding criteria (Ferri et al., 2017). The index of periodic limb movements corresponds to the total number of periodic limb movements divided by the sleep period in hours. Periodic limb movement syndrome is considered present when the periodic limb movement index is ≥15/hour (Haba-Rubio et al., 2018). Diagnoses of restless legs syndrome are based on the diagnostic criteria established by the International Restless Legs Syndrome Study Group (Allen et al., 2014). Insomnia disorders are diagnosed according to the criteria of the American Academy of Sleep Medicine (Edinger et al., 2004), and sleep deprivation is defined as a sleep duration of less than 6 hours (Hein et al., 2019).

S3/Detailed analyses for each multivariate model tested

S2.1/Model 1

```
. xi: logit Groupe_ANH i.Groupe_CRP1 i.Groupe_sexe i.Groupe_age i.Groupe_ATD i.Groupe_psycho, or
i.Groupe_CRP1     _IGroupe_CR_0-1     (naturally coded; _IGroupe_CR_0 omitted)
i.Groupe_sexe     _IGroupe_se_0-1     (naturally coded; _IGroupe_se_0 omitted)
i.Groupe_age      _IGroupe_ag_0-1     (naturally coded; _IGroupe_ag_0 omitted)
i.Groupe_ATD      _IGroupe_AT_0-1     (naturally coded; _IGroupe_AT_0 omitted)
i.Groupe_psycho   _IGroupe_ps_0-1     (naturally coded; _IGroupe_ps_0 omitted)

Iteration 0:   log likelihood = -334.05555
Iteration 1:   log likelihood =  -310.2628
Iteration 2:   log likelihood = -310.18924
Iteration 3:   log likelihood = -310.18923

Logistic regression                               Number of obs     =        496
                                                  LR chi2(5)        =      47.73
                                                  Prob > chi2       =     0.0000
Log likelihood = -310.18923                       Pseudo R2         =     0.0714
```

Groupe_ANH	Odds Ratio	Std. Err.	z	P>|z|	[95% Conf.	Interval]
_IGroupe_CR_1	1.734061	.363523	2.63	0.009	1.149797	2.615216
_IGroupe_se_1	.8181795	.1623884	-1.01	0.312	.5545058	1.207233
_IGroupe_ag_1	1.233011	.2536959	1.02	0.309	.8238147	1.845459
_IGroupe_AT_1	2.627434	.5737257	4.42	0.000	1.712631	4.030881
_IGroupe_ps_1	1.678727	.4093981	2.12	0.034	1.040864	2.707485
_cons	.3807117	.0681873	-5.39	0.000	.2680045	.5408171

```
. estat gof, group(10)

Logistic model for Groupe_ANH, goodness-of-fit test

  (Table collapsed on quantiles of estimated probabilities)
  (There are only 9 distinct quantiles because of ties)

       number of observations =       496
             number of groups =         9
      Hosmer-Lemeshow chi2(7) =         7.99
                  Prob > chi2 =         0.3334

. linktest

Iteration 0:   log likelihood = -334.05555
Iteration 1:   log likelihood = -309.49255
Iteration 2:   log likelihood = -309.40597
Iteration 3:   log likelihood = -309.40592
Iteration 4:   log likelihood = -309.40592

Logistic regression                               Number of obs     =        496
                                                  LR chi2(2)        =      49.30
                                                  Prob > chi2       =     0.0000
Log likelihood = -309.40592                       Pseudo R2         =     0.0738
```

Groupe_ANH	Coef.	Std. Err.	z	P>|z|	[95% Conf.	Interval]
_hat	1.117882	.1864238	6.00	0.000	.7524978	1.483265
_hatsq	.2935249	.2389946	1.23	0.219	-.174896	.7619458
_cons	-.1157702	.1471558	-0.79	0.431	-.4041903	.1726499

```
. collin Groupe_CRP1 Groupe_sexe Groupe_age Groupe_ATD Groupe_psycho
(obs=496)

  Collinearity Diagnostics

                        SQRT                   R-
  Variable      VIF     VIF    Tolerance    Squared
----------------------------------------------------
Groupe_CRP1      1.03    1.02    0.9675      0.0325
Groupe_sexe      1.04    1.02    0.9589      0.0411
Groupe_age      1.03    1.02    0.9692      0.0308
Groupe_ATD      1.14    1.07    0.8782      0.1218
Groupe_psycho      1.14    1.07    0.8776      0.1224
----------------------------------------------------
  Mean VIF      1.08
```

```
. bootstrap, reps(1000): logit Groupe_ANH i.Groupe_CRP1 i.Groupe_sexe i.Groupe_age i.Groupe_ATD i.Groupe_psycho
> , or
(running logit on estimation sample)

Bootstrap replications (1000)
────┼─── 1 ───┼─── 2 ───┼─── 3 ───┼─── 4 ───┼─── 5
..................................................    50
..................................................   100
..................................................   150
..................................................   200
..................................................   250
..................................................   300
..................................................   350
..................................................   400
..................................................   450
..................................................   500
..................................................   550
..................................................   600
..................................................   650
..................................................   700
..................................................   750
..................................................   800
..................................................   850
..................................................   900
..................................................   950
..................................................  1000

Logistic regression                               Number of obs     =        496
                                                  Replications      =      1,000
                                                  Wald chi2(5)      =      40.66
                                                  Prob > chi2       =     0.0000
Log likelihood = -310.18923                       Pseudo R2         =     0.0714
```

Groupe_ANH	Observed Odds Ratio	Bootstrap Std. Err.	z	P>|z|	Normal-based [95% Conf.	Interval]
1.Groupe_CRP1	1.734061	.3678584	2.59	0.009	1.144177	2.628062
1.Groupe_sexe	.8181795	.1644862	-1.00	0.318	.5517263	1.213315
1.Groupe_age	1.233011	.2494331	1.04	0.300	.8294158	1.832996
1.Groupe_ATD	2.627434	.582581	4.36	0.000	1.701355	4.057596
1.Groupe_psycho	1.678727	.4041078	2.15	0.031	1.047313	2.690814
_cons	.3807117	.0722648	-5.09	0.000	.2624372	.5522898

S2.2/Model 2

```
. xi: logit Groupe_ANH i.Groupe_CRP1 i.Groupe_sexe i.Groupe_age i.Groupe_ATD i.Groupe_psycho i.Groupe_diabete ,
>  or
i.Groupe_CRP1     _IGroupe_CR_0-1     (naturally coded; _IGroupe_CR_0 omitted)
i.Groupe_sexe     _IGroupe_se_0-1     (naturally coded; _IGroupe_se_0 omitted)
i.Groupe_age      _IGroupe_ag_0-1     (naturally coded; _IGroupe_ag_0 omitted)
i.Groupe_ATD      _IGroupe_AT_0-1     (naturally coded; _IGroupe_AT_0 omitted)
i.Groupe_psycho   _IGroupe_ps_0-1     (naturally coded; _IGroupe_ps_0 omitted)
i.Groupe_diab~e   _IGroupe_di_0-1     (naturally coded; _IGroupe_di_0 omitted)

Iteration 0:   log likelihood = -334.05555
Iteration 1:   log likelihood = -305.59395
Iteration 2:   log likelihood = -305.46693
Iteration 3:   log likelihood = -305.46689
Iteration 4:   log likelihood = -305.46689

Logistic regression                               Number of obs     =        496
                                                  LR chi2(6)        =      57.18
                                                  Prob > chi2       =     0.0000
Log likelihood = -305.46689                       Pseudo R2         =     0.0856
```

Groupe_ANH	Odds Ratio	Std. Err.	z	P>|z|	[95% Conf.	Interval]
_IGroupe_CR_1	1.645258	.3497416	2.34	0.019	1.08465	2.495621
_IGroupe_se_1	.7800786	.1570847	-1.23	0.217	.5256923	1.157564
_IGroupe_ag_1	1.064153	.2276859	0.29	0.771	.6996508	1.618553
_IGroupe_AT_1	2.488229	.5506317	4.12	0.000	1.61259	3.839341
_IGroupe_ps_1	1.748392	.4309634	2.27	0.023	1.078513	2.834345
_IGroupe_di_1	2.338383	.6508279	3.05	0.002	1.355208	4.034831
_cons	.366296	.0663758	-5.54	0.000	.2567957	.5224886

```
. estat gof, group(10)
```

Logistic model for Groupe_ANH, goodness-of-fit test

```
  (Table collapsed on quantiles of estimated probabilities)

       number of observations =       496
             number of groups =        10
      Hosmer-Lemeshow chi2(8) =         8.95
                  Prob > chi2 =         0.3464

. linktest

Iteration 0:   log likelihood = -334.05555
Iteration 1:   log likelihood = -304.98758
Iteration 2:   log likelihood = -304.81349
Iteration 3:   log likelihood = -304.81339
Iteration 4:   log likelihood = -304.81339

Logistic regression                               Number of obs     =        496
                                                  LR chi2(2)        =      58.48
                                                  Prob > chi2       =     0.0000
Log likelihood = -304.81339                       Pseudo R2         =     0.0875
```

Groupe_ANH	Coef.	Std. Err.	z	P>|z|	[95% Conf.	Interval]
_hat	.9506093	.1434001	6.63	0.000	.6695503	1.231668
_hatsq	-.1868039	.1603625	-1.16	0.244	-.5011087	.1275009
_cons	.0981074	.1405901	0.70	0.485	-.1774441	.3736589

```
. collin Groupe_CRP1 Groupe_sexe Groupe_age Groupe_ATD Groupe_psycho Groupe_diabete
(obs=496)

  Collinearity Diagnostics

                        SQRT                   R-
  Variable      VIF     VIF    Tolerance    Squared
----------------------------------------------------
Groupe_CRP1      1.04    1.02    0.9573      0.0427
Groupe_sexe      1.05    1.02    0.9541      0.0459
Groupe_age      1.09    1.04    0.9190      0.0810
Groupe_ATD      1.15    1.07    0.8672      0.1328
Groupe_psycho      1.14    1.07    0.8764      0.1236
Groupe_diabete      1.09    1.04    0.9172      0.0828
----------------------------------------------------
  Mean VIF      1.09
```

```
. bootstrap, reps(1000): logit Groupe_ANH i.Groupe_CRP1 i.Groupe_sexe i.Groupe_age i.Groupe_ATD i.Groupe_psycho
>  i.Groupe_diabete , or
(running logit on estimation sample)

Bootstrap replications (1000)
----+--- 1 ---+--- 2 ---+--- 3 ---+--- 4 ---+--- 5
..................................................    50
..................................................   100
..................................................   150
..................................................   200
..................................................   250
..................................................   300
..................................................   350
..................................................   400
..................................................   450
..................................................   500
..................................................   550
..................................................   600
..................................................   650
..................................................   700
..................................................   750
..................................................   800
..................................................   850
..................................................   900
..................................................   950
..................................................  1000

Logistic regression                             Number of obs     =        496
                                                Replications      =      1,000
                                                Wald chi2(6)      =      42.04
                                                Prob > chi2       =     0.0000
Log likelihood = -305.46689                     Pseudo R2         =     0.0856

------------------------------------------------------------------------------------
                 |   Observed   Bootstrap                         Normal-based
      Groupe_ANH | Odds Ratio   Std. Err.      z    P>|z|     [95% Conf. Interval]
-----------------+------------------------------------------------------------------
   1.Groupe_CRP1 |   1.645258   .3552544     2.31   0.021      1.07755    2.512065
   1.Groupe_sexe |   .7800786   .1578828    -1.23   0.220     .5246391    1.159888
    1.Groupe_age |   1.064153   .2312116     0.29   0.775     .6951223    1.629098
    1.Groupe_ATD |   2.488229   .5489253     4.13   0.000     1.614759    3.834184
 1.Groupe_psycho |   1.748392   .4465156     2.19   0.029     1.059873    2.884192
1.Groupe_diabete |   2.338383   .7420269     2.68   0.007     1.255475    4.355349
           _cons |    .366296   .0684189    -5.38   0.000     .2540036    .5282319
------------------------------------------------------------------------------------
```

S2.3/Model 3

```
. xi: logit Groupe_ANH i.Groupe_CRP1 i.Groupe_sexe i.Groupe_age i.Groupe_ATD i.Groupe_psycho i.Groupe_diabete i
> .Groupe_insomnie i.Groupe_EPW , or
i.Groupe_CRP1     _IGroupe_CR_0-1     (naturally coded; _IGroupe_CR_0 omitted)
i.Groupe_sexe     _IGroupe_se_0-1     (naturally coded; _IGroupe_se_0 omitted)
i.Groupe_age      _IGroupe_ag_0-1     (naturally coded; _IGroupe_ag_0 omitted)
i.Groupe_ATD      _IGroupe_AT_0-1     (naturally coded; _IGroupe_AT_0 omitted)
i.Groupe_psycho   _IGroupe_ps_0-1     (naturally coded; _IGroupe_ps_0 omitted)
i.Groupe_diab~e   _IGroupe_di_0-1     (naturally coded; _IGroupe_di_0 omitted)
i.Groupe_inso~e   _IGroupe_in_0-1     (naturally coded; _IGroupe_in_0 omitted)
i.Groupe_EPW      _IGroupe_EP_0-1     (naturally coded; _IGroupe_EP_0 omitted)

Iteration 0:   log likelihood = -334.05555
Iteration 1:   log likelihood = -301.38917
Iteration 2:   log likelihood = -301.30174
Iteration 3:   log likelihood = -301.30174

Logistic regression                               Number of obs     =        496
                                                  LR chi2(8)        =      65.51
                                                  Prob > chi2       =     0.0000
Log likelihood = -301.30174                       Pseudo R2         =     0.0980
```

Groupe_ANH	Odds Ratio	Std. Err.	z	P>|z|	[95% Conf.	Interval]
_IGroupe_CR_1	1.710764	.3690634	2.49	0.013	1.120883	2.611079
_IGroupe_se_1	.7968595	.1620956	-1.12	0.264	.5348499	1.187221
_IGroupe_ag_1	1.124723	.2457915	0.54	0.591	.7328715	1.726088
_IGroupe_AT_1	2.367851	.5297344	3.85	0.000	1.527299	3.671003
_IGroupe_ps_1	1.728803	.4335601	2.18	0.029	1.057489	2.82628
_IGroupe_di_1	2.320699	.6530573	2.99	0.003	1.336861	4.028576
_IGroupe_in_1	1.413227	.3260323	1.50	0.134	.8991649	2.221185
_IGroupe_EP_1	1.806202	.4516761	2.36	0.018	1.106386	2.948668
_cons	.2487404	.0656262	-5.27	0.000	.14831	.4171788

```
. estat gof, group(10)
```

Logistic model for Groupe_ANH, goodness-of-fit test

```
  (Table collapsed on quantiles of estimated probabilities)

       number of observations =       496
             number of groups =        10
      Hosmer-Lemeshow chi2(8) =         7.83
                  Prob > chi2 =         0.4505

. linktest

Iteration 0:   log likelihood = -334.05555
Iteration 1:   log likelihood = -300.60227
Iteration 2:   log likelihood = -300.40795
Iteration 3:   log likelihood = -300.40762
Iteration 4:   log likelihood = -300.40762

Logistic regression                               Number of obs     =        496
                                                  LR chi2(2)        =      67.30
                                                  Prob > chi2       =     0.0000
Log likelihood = -300.40762                       Pseudo R2         =     0.1007
```

Groupe_ANH	Coef.	Std. Err.	z	P>|z|	[95% Conf.	Interval]
_hat	.9590491	.133	7.21	0.000	.6983738	1.219724
_hatsq	-.1741892	.1266853	-1.37	0.169	-.4224878	.0741095
_cons	.1036201	.135167	0.77	0.443	-.1613024	.3685426

```
. collin Groupe_CRP1 Groupe_sexe Groupe_age Groupe_ATD Groupe_psycho Groupe_diabete Groupe_insomnie Groupe_EPW
(obs=496)

  Collinearity Diagnostics
```

Variable	VIF	SQRT VIF	Tolerance	R-Squared
Groupe_CRP1	1.05	1.02	0.9520	0.0480
Groupe_sexe	1.05	1.03	0.9497	0.0503
Groupe_age	1.11	1.06	0.8970	0.1030
Groupe_ATD	1.16	1.08	0.8584	0.1416
Groupe_psycho	1.16	1.08	0.8635	0.1365
Groupe_diabete	1.09	1.05	0.9150	0.0850
Groupe_insomnie	1.06	1.03	0.9425	0.0575
Groupe_EPW	1.01	1.01	0.9893	0.0107
Mean VIF	1.09			

```
. bootstrap, reps(1000): logit Groupe_ANH i.Groupe_CRP1 i.Groupe_sexe i.Groupe_age i.Groupe_ATD i.Groupe_psycho
>  i.Groupe_diabete i.Groupe_insomnie i.Groupe_EPW , or
(running logit on estimation sample)

Bootstrap replications (1000)
----+--- 1 ---+--- 2 ---+--- 3 ---+--- 4 ---+--- 5
..................................................    50
..................................................   100
..................................................   150
..................................................   200
..................................................   250
..................................................   300
..................................................   350
..................................................   400
..................................................   450
..................................................   500
..................................................   550
..................................................   600
..................................................   650
..................................................   700
..................................................   750
..................................................   800
..................................................   850
..................................................   900
..................................................   950
..................................................  1000

Logistic regression                             Number of obs     =        496
                                                Replications      =      1,000
                                                Wald chi2(8)      =      51.15
                                                Prob > chi2       =     0.0000
Log likelihood = -301.30174                     Pseudo R2         =     0.0980
```

Groupe_ANH	Observed Odds Ratio	Bootstrap Std. Err.	z	P>|z|	Normal-based [95% Conf.	Interval]
1.Groupe_CRP1	1.710764	.3756897	2.45	0.014	1.112406	2.630976
1.Groupe_sexe	.7968595	.1628675	-1.11	0.267	.5338354	1.189477
1.Groupe_age	1.124723	.245835	0.54	0.591	.7328159	1.726219
1.Groupe_ATD	2.367851	.5516867	3.70	0.000	1.499797	3.738318
1.Groupe_psycho	1.728803	.4548273	2.08	0.037	1.032297	2.895252
1.Groupe_diabete	2.320699	.7326626	2.67	0.008	1.249937	4.308735
1.Groupe_insomnie	1.413227	.3353918	1.46	0.145	.8875688	2.250204
1.Groupe_EPW	1.806202	.4632966	2.30	0.021	1.092522	2.986085
_cons	.2487404	.0661496	-5.23	0.000	.1476996	.4189031

S2.4/Model 4

```
. xi: logit Groupe_ANH i.Groupe_CRP1 i.Groupe_sexe i.Groupe_age i.Groupe_ATD i.Groupe_psycho i.Groupe_diabete i
> .Groupe_insomnie i.Groupe_EPW i.Groupe_spiel i.Groupe_BECK i.MDD_duration , or
i.Groupe_CRP1     _IGroupe_CR_0-1     (naturally coded; _IGroupe_CR_0 omitted)
i.Groupe_sexe     _IGroupe_se_0-1     (naturally coded; _IGroupe_se_0 omitted)
i.Groupe_age      _IGroupe_ag_0-1     (naturally coded; _IGroupe_ag_0 omitted)
i.Groupe_ATD      _IGroupe_AT_0-1     (naturally coded; _IGroupe_AT_0 omitted)
i.Groupe_psycho   _IGroupe_ps_0-1     (naturally coded; _IGroupe_ps_0 omitted)
i.Groupe_diab~e   _IGroupe_di_0-1     (naturally coded; _IGroupe_di_0 omitted)
i.Groupe_inso~e   _IGroupe_in_0-1     (naturally coded; _IGroupe_in_0 omitted)
i.Groupe_EPW      _IGroupe_EP_0-1     (naturally coded; _IGroupe_EP_0 omitted)
i.Groupe_spiel    _IGroupe_sp_0-3     (naturally coded; _IGroupe_sp_0 omitted)
i.Groupe_BECK     _IGroupe_BE_0-1     (naturally coded; _IGroupe_BE_0 omitted)
i.MDD_duration    _IMDD_durat_0-1     (naturally coded; _IMDD_durat_0 omitted)

Iteration 0:   log likelihood = -334.05555
Iteration 1:   log likelihood =  -284.6223
Iteration 2:   log likelihood = -284.38411
Iteration 3:   log likelihood = -284.38396
Iteration 4:   log likelihood = -284.38396

Logistic regression                               Number of obs     =        496
                                                  LR chi2(13)       =      99.34
                                                  Prob > chi2       =     0.0000
Log likelihood = -284.38396                       Pseudo R2         =     0.1487

------------------------------------------------------------------------------
   Groupe_ANH | Odds Ratio   Std. Err.      z    P>|z|     [95% Conf. Interval]
--------------+---------------------------------------------------------------
_IGroupe_CR_1 |   1.769004   .3997498     2.52   0.012     1.136001    2.754727
_IGroupe_se_1 |    .886704   .1889774    -0.56   0.573     .5839393    1.346448
_IGroupe_ag_1 |   1.304477    .298289     1.16   0.245     .8332899      2.0421
_IGroupe_AT_1 |   2.414581    .951657     2.24   0.025     1.115215    5.227875
_IGroupe_ps_1 |   1.738366   .4816484     2.00   0.046     1.009949     2.99215
_IGroupe_di_1 |   2.224229   .6566411     2.71   0.007     1.247057    3.967097
_IGroupe_in_1 |    1.13137    .274604     0.51   0.611     .7030767    1.820567
_IGroupe_EP_1 |   1.605245   .4184118     1.82   0.069     .9631038    2.675528
_IGroupe_sp_1 |   1.667956   .5803281     1.47   0.141      .843389    3.298688
_IGroupe_sp_2 |   .7653889   .2519403    -0.81   0.417     .4015089    1.459047
_IGroupe_sp_3 |   2.423469   .6545038     3.28   0.001     1.427429    4.114534
_IGroupe_BE_1 |    1.74472   .4418642     2.20   0.028     1.062068    2.866153
_IMDD_durat_1 |   .7882991   .3078112    -0.61   0.542     .3667059    1.694588
        _cons |   .1471866   .0477605    -5.90   0.000     .0779225    .2780186
------------------------------------------------------------------------------

. estat gof, group(10)

Logistic model for Groupe_ANH, goodness-of-fit test

  (Table collapsed on quantiles of estimated probabilities)

       number of observations =       496
             number of groups =        10
      Hosmer-Lemeshow chi2(8) =         9.11
                  Prob > chi2 =         0.3329

. linktest

Iteration 0:   log likelihood = -334.05555
Iteration 1:   log likelihood = -284.38362
Iteration 2:   log likelihood = -284.06145
Iteration 3:   log likelihood = -284.06094
Iteration 4:   log likelihood = -284.06094

Logistic regression                               Number of obs     =        496
                                                  LR chi2(2)        =      99.99
                                                  Prob > chi2       =     0.0000
Log likelihood = -284.06094                       Pseudo R2         =     0.1497

------------------------------------------------------------------------------
  Groupe_ANH |      Coef.   Std. Err.      z    P>|z|     [95% Conf. Interval]
-------------+----------------------------------------------------------------
        _hat |   .9701609   .1164076     8.33   0.000     .7420063    1.198316
      _hatsq |  -.0771173   .0943022    -0.82   0.413    -.2619461    .1077115
       _cons |   .0617334    .133902     0.46   0.645    -.2007097    .3241765
------------------------------------------------------------------------------

. collin Groupe_CRP1 Groupe_sexe Groupe_age Groupe_ATD Groupe_psycho Groupe_diabete Groupe_insomnie Groupe_EPW
> Groupe_spiel Groupe_BECK MDD_duration
(obs=496)

  Collinearity Diagnostics

                        SQRT                   R-
  Variable      VIF     VIF    Tolerance    Squared
----------------------------------------------------
Groupe_CRP1      1.05    1.03    0.9505      0.0495
Groupe_sexe      1.07    1.04    0.9313      0.0687
Groupe_age      1.13    1.06    0.8869      0.1131
Groupe_ATD      3.17    1.78    0.3154      0.6846
Groupe_psycho      1.29    1.13    0.7769      0.2231
Groupe_diabete      1.11    1.05    0.9045      0.0955
Groupe_insomnie      1.10    1.05    0.9109      0.0891
Groupe_EPW      1.03    1.01    0.9747      0.0253
Groupe_spiel      1.28    1.13    0.7788      0.2212
Groupe_BECK      1.32    1.15    0.7599      0.2401
MDD_duration      3.46    1.86    0.2887      0.7113
----------------------------------------------------
  Mean VIF      1.55
```

```
. bootstrap, reps(1000): logit Groupe_ANH i.Groupe_CRP1 i.Groupe_sexe i.Groupe_age i.Groupe_ATD i.Groupe_psycho
>  i.Groupe_diabete i.Groupe_insomnie i.Groupe_EPW i.Groupe_spiel i.Groupe_BECK i.MDD_duration , or
(running logit on estimation sample)

Bootstrap replications (1000)
----+--- 1 ---+--- 2 ---+--- 3 ---+--- 4 ---+--- 5
..................................................    50
..................................................   100
..................................................   150
..................................................   200
..................................................   250
..................................................   300
..................................................   350
..................................................   400
..................................................   450
..................................................   500
..................................................   550
..................................................   600
..................................................   650
..................................................   700
..................................................   750
..................................................   800
..................................................   850
..................................................   900
..................................................   950
..................................................  1000

Logistic regression                             Number of obs     =        496
                                                Replications      =      1,000
                                                Wald chi2(13)     =      70.15
                                                Prob > chi2       =     0.0000
Log likelihood = -284.38396                     Pseudo R2         =     0.1487
```

Groupe_ANH	Observed Odds Ratio	Bootstrap Std. Err.	z	P>|z|	Normal-based [95% Conf.	Interval]
1.Groupe_CRP1	1.769004	.4431516	2.28	0.023	1.082667	2.89043
1.Groupe_sexe	.886704	.1978254	-0.54	0.590	.5726298	1.373041
1.Groupe_age	1.304477	.3206511	1.08	0.280	.8057575	2.111878
1.Groupe_ATD	2.414581	1.081025	1.97	0.049	1.004044	5.80672
1.Groupe_psycho	1.738366	.5323042	1.81	0.071	.9538836	3.168015
1.Groupe_diabete	2.224229	.7718463	2.30	0.021	1.126672	4.390978
1.Groupe_insomnie	1.13137	.2938542	0.48	0.635	.6800167	1.882304
1.Groupe_EPW	1.605245	.4443104	1.71	0.087	.9331255	2.761484
Groupe_spiel						
1	1.667956	.6432772	1.33	0.185	.7832554	3.551941
2	.7653889	.2722491	-0.75	0.452	.3811618	1.536933
3	2.423469	.6911401	3.10	0.002	1.385755	4.238269
1.Groupe_BECK	1.74472	.4590929	2.12	0.034	1.04171	2.922165
1.MDD_duration	.7882991	.3538693	-0.53	0.596	.3270278	1.900192
_cons	.1471866	.0500413	-5.64	0.000	.0755914	.2865921

References:

AASM Scoring Manual—American Academy of Sleep Medicine. (n.d.). *American Academy of Sleep Medicine – Association for Sleep Clinicians and Researchers*. Retrieved August 25, 2025, from https://aasm.org/clinical-resources/scoring-manual/

Berry, R. B., Budhiraja, R., Gottlieb, D. J., Gozal, D., Iber, C., Kapur, V. K., Marcus, C. L., Mehra, R., Parthasarathy, S., Quan, S. F., Redline, S., Strohl, K. P., Ward, S. L. D., & Tangredi, M. M. (n.d.). Rules for Scoring Respiratory Events in Sleep: Update of the 2007 AASM Manual for the Scoring of Sleep and Associated Events. *Journal of Clinical Sleep Medicine*, *08*(05), 597–619. https://doi.org/10.5664/jcsm.2172

Edinger, J. D., Bonnet, M. H., Bootzin, R. R., Doghramji, K., Dorsey, C. M., Espie, C. A., Jamieson, A. O., McCall, W. V., Morin, C. M., Stepanski, E. J., & American Academy of Sleep Medicine Work Group. (2004). Derivation of research diagnostic criteria for insomnia: Report of an American Academy of Sleep Medicine Work Group. *Sleep*, *27*(8), 1567–1596. https://doi.org/10.1093/sleep/27.8.1567

Ferri, R., Koo, B. B., Picchietti, D. L., & Fulda, S. (2017). Periodic leg movements during sleep: Phenotype, neurophysiology, and clinical significance. *Sleep Medicine*, *31*, 29–38. https://doi.org/10.1016/j.sleep.2016.05.014

Fleetham, J., Ayas, N., Bradley, D., Ferguson, K., Fitzpatrick, M., George, C., Hanly, P., Hill, F., Kimoff, J., Kryger, M., Morrison, D., Series, F., Tsai, W., & CTS Sleep Disordered Breathing Committee. (2006). Canadian Thoracic Society guidelines: Diagnosis and treatment of sleep disordered breathing in adults. *Canadian Respiratory Journal*, *13*(7), 387–392. https://doi.org/10.1155/2006/627096

Haba-Rubio, J., Marti-Soler, H., Tobback, N., Andries, D., Marques-Vidal, P., Vollenweider, P., Preisig, M., & Heinzer, R. (2018). Clinical significance of periodic limb movements during sleep: The HypnoLaus study. *Sleep Medicine*, *41*, 45–50. https://doi.org/10.1016/j.sleep.2017.09.014

Hein, M., Lanquart, J.-P., Loas, G., Hubain, P., & Linkowski, P. (2019). Risk of high blood pressure associated with objective insomnia and self-reported insomnia complaints in major depression: A study on 703 individuals. *Clinical and Experimental Hypertension*, *41*(6), 538–547. https://doi.org/10.1080/10641963.2018.1516775

Kushida, C. A., Littner, M. R., Morgenthaler, T., Alessi, C. A., Bailey, D., Coleman, J., Jr., Friedman, L., Hirshkowitz, M., Kapen, S., Kramer, M., Lee-Chiong, T., Loube, D. L., Owens, J., Pancer, J. P., & Wise, M. (2005). Practice Parameters for the Indications for Polysomnography and Related Procedures: An Update for 2005. *Sleep*, *28*(4), 499–523. https://doi.org/10.1093/sleep/28.4.499
